# *Gardenia jasminoides* Ellis Fruit Extracts Attenuated Colitis in 2,4,6-Trinitrobenzenesulfonic Acid-Induced Rats

**DOI:** 10.1155/2021/9920379

**Published:** 2021-12-15

**Authors:** Jing Liu, Chao Yang, Zhigui Wu, Jianguo Pei, Yao Chen, Xiao Huang, Sha Gao, Rui Kan, Wenna Zhang, Saisai Xie, Xiaomei Fu

**Affiliations:** ^1^School of Pharmacy, Jiangxi University of Chinese Medicine, Nanchang 330006, China; ^2^FAN Cuisheng Studio of National Famous TCM, Nanchang 330006, China; ^3^School of Life Sciences, Anhui University, Hefei 230601, Anhui, China; ^4^National Pharmaceutical Engineering Center for Solid Preparation in Chinese Herbal Medicine, Jiangxi University of Chinese Medicine, Nanchang 330006, China

## Abstract

Ulcerative colitis (UC) is a relapsing inflammatory disease with an unknown precise etiology. The purpose of this study is to investigate the protective effects of *Gardenia jasminoides* Ellis fruit extracts (GFE) on 2,4,6-trinitrobenzenesulfonic acid (TNBS)-induced colitis in rats. GFE (50 mg/kg, 100 mg/kg) were administered orally for 7 days after induction. Meanwhile, the chemical components of GFE were performed by UPLC-QTOF-MS/MS. GFE significantly decreased DAI scores and ameliorated macroscopic and histologic damage. It also reduced the levels of MPO, NO, MDA, IL-1*β*, TNF-*α*, and IL-6, while increasing the level of SOD. Moreover, 56 components were identified in GFE using a UPLC-QTOF-MS/MS method, which can be categorized into six structural groups. Our results indicated that GFE has an ameliorative effect on TNBS-induced colitis in rats, which may further verify its anti-inflammatory and antioxidative properties. Therefore, GFE can be a promising protective agent of colitis that deserves further investigation.

## 1. Introduction

Ulcerative colitis (UC) is a relapsing inflammatory disease that affects the mucosal layer of the rectum and colon. The symptoms of UC include chronic diarrhea, stomach pain, blood in stool, and weight loss in the clinic [1, 2]. It was found that UC occurs more and more commonly and worldwide in recent years, and the long-term abdominal discomfort already lowers the life quality of patients. Meanwhile, UC is widely accepted as a risk factor for colorectal cancer according to the clinical data [3]. Hence, World Health Organization lists UC as a miscellaneous problem.

Despite the precise cause of UC has not yet been completely elucidated, it is wildly considered that proinflammatory cytokines such as interleukin-1*β* (1L-1*β*), tumor necrosis factor-*α* (TNF-*α*), and interleukin-6 (1L-6) play an essential role in the process of the pathogenesis and progression of UC [4, 5]. Current treatments of UC mainly rely on anti-inflammatory medicines such as aminosalicylates and immunosuppressive and corticosteroid agents. However, most of these drugs have lots of unacceptable adverse effects, such as poor tolerance and high recurrence rate, which are not quite suitable for long-term treatment [6]. Therefore, it is still urgent to search new safe and effective agents for adjunct therapies.

Recently, more and more studies have focused on natural products as alternative or complimentary medicines for UC treatment. A series of natural products had been confirmed to be able to treat UC through anti-inflammatory actions, such as *Forsythia suspensa* Fructus, *Eucommia ulmoides* leaf, and brusatol, which can potentially enlarge the scope of drug candidates for UC treatment as well [7–9].

*Gardenia jasminoides* Ellis is a flowering plant belonging to Rubiaceae family. Its fruit has been used as food coloring in oriental countries [10]. As a natural plant, the fruit is used not only as a food but also as a medicine to treat hepatic disorders, headache, jaundice, inflammation, and hypertension [11–13]. Moreover, many bioactive ingredients from *Gardenia jasminoides* Ellis fruit extracts (GFE) such as iridoids, crocins, and organic acids have also shown anti-inflammatory effects. Specifically, geniposide and chlorogenic acid have shown good protective effects in rat colitis models [14, 15]. Taken together, GFE has potential for the treatment of UC.

Therefore, the present study is to investigate whether GFE could have a protective effect in the rat colitis model induced by trinitrobenzene sulphonic acid (TNBS) and its mechanisms associated and to identify its bioactive ingredients by UPLC-QTOF-MS/MS.

## 2. Materials and Methods

### 2.1. Chemicals, Reagents, and Plant Material

The fruits of *Gardenia jasminoides* Ellis were collected from the GAP Base District of Jiangxi Province, China, and identified by Professor Cuisheng Fan, College of Pharmacy of Jiangxi University of Traditional Chinese Medicine. A voucher specimen (GJ-181027) was deposited at the department of identificology of Chinese Materia Medica of Jiangxi University of Traditional Chinese Medicine. TNBS was obtained from Sigma-Aldrich, USA. Sulfasalazine (SASP) was obtained from Shanghai Xinyi Tianping Pharmaceutical, China. Myeloperoxidase (MPO) determination kit was obtained from Jiancheng Bioengineering Institute, China; nitric oxide (NO), malondialdehyde (MDA), and superoxide dismutase (SOD) assay kits were obtained from Beyotime Institute of Biotechnology, China. The interleukin-1*β* (IL-1*β*), necrosis factor-*α* (TNF-*α*), and interleukin-6 (IL-6) ELISA kits were obtained from MultiSciences (Lianke) Biotech, China. Acetonitrile, methanol, and formic acid were all of HPLC grade and were purchased from Merck, Germany. Ultrapure water was prepared by a Milli-Q system (Millipore, Bedford, USA).

### 2.2. Preparation of GFE

The fruits (1500°g) were crushed, extracted with 70% aqueous ethanol for 2 hours under reflux, and extracted twice. The extract was concentrated by rotary evaporator and dried with lyophilizer. The yield of the extract was 21.39% (w/w). The dried extract was kept at −20°C.

### 2.3. UPLC-QTOF-MS/MS Analysis

The chemical compositions of GFE were analysed by UPLC-QTOF-MS/MS. The dried extract (1 mg) was dissolved in 1 mL acetonitrile and then filtered through a 0.22 *μ*m membrane filter. UPLC analysis was performed with a Shimadzu Prominence UPLC system (Nexera UHPLC LC-30A, Kyoto, Japan) comprised of a LC-30A binary pump, a LC-30AD solvent delivery system, a SIL-30AC autosampler, and a CTO-30AC column oven. Separation was carried on an ACE Excel Super C18 analytical column (2.1 × 50 mm, 1.7 *μ*m) at 40 °C. The mobile phase consisted of (*A*) water-0.1% formic acid and (B) acetonitrile: 0–10 min, 5–15% B; 10–12 min, 15–21% B; 12–15 min, 21% B; 15–18 min, 21–23% B; 18–20 min, 23–50% B; 20–30 min, 50–95% B; 30–32 min, 95% B. 3 *μ*l sample solution was injected and the flow rate was 0.3 mL/min.

The mass data were recorded on a triple TOF 5600^+^ mass spectrometer (AB Sciex, USA), equipped with UPLC system. Samples were analysed in negative ion mode and the mass scan range was set from m/*z* 200 to m/*z* 1000. The other operation parameters in negative mode were optimized as follows: ion spray voltage, −4500 V; ion source temperature, 500°C; nebulizer gas (GS1), 50 psi; curtain gas pressure, 40 psi; heater gas (GS2), 50 psi; declustering potential, −100 V; collision energy, −45 eV; and collision energy spread, (±) 15 eV. All data acquisition and analysis were used by the PeakView 1.2 software (AB Sciex, USA).

### 2.4. Animals and Treatment

Healthy male Sprague-Dawley (SD) rats (180–220 g) were purchased from Hunan SJA Laboratory Animal Co., Ltd. (Hunan, China; Certificate of Conformity: SCXK2013-0004). Animals were housed in polypropylene cages at 22 ± 2°C, under 12 h day/night cycles. Rats were allowed free access to food and water. All procedures and experimental protocols were approved by Institutional Animals Ethics Committee of Jiangxi University of Traditional Chinese Medicine (certificate number: 201901112) and were carried out according to international standards and ethical guidelines on animal welfare.

Rat model of colitis was produced following the method described previously [16]. A catheter was inserted into the rat colon and then TNBS-ethanol solution (50%, v/v) was slowly administered at a dose of 0.5 mL/kg though the catheter. The control group of rats was treated with 50% ethanol solution. All rats were kept in a vertical position for 30 s to prevent from leaking out before returning to their cage. Rats were distributed into five groups of seven rats each: control group, TNBS group, SASP group (500 mg/kg), and GFE treated groups (50 mg/kg, 100 mg/kg). The TNBS and control groups were administered orally with 0.9% saline at the same volume. All drugs were administered orally for 7 consecutive days after induction. Rats were sacrificed under anesthesia to collect specimens on the 8th day.

### 2.5. Disease Activity Index Evaluation

The rats' body weight, stool occult, and stool formation were recorded daily to calculate disease activity index (DAI) after modeling (Day 2). DAI was determined according to the previously scoring system [17].

### 2.6. Macroscopic Scoring of Colitis

After sacrificing the rats, the colons were removed and then flushed with ice-cold normal saline for macroscopic observation. The macroscopic scores were determined according to the criteria described in previous studies with a total score of 4 [18] (Table. 1).

### 2.7. Histological Assessment of Colitis

After the macroscopic assessment, about 0.5 × 0.5 cm^2^ sections of colon tissues were fixed in 4% formalin, embedded in paraffin, and then stained with hematoxylin and eosin (H&E). The histopathological scores were assessed by the previous standard with a total score of 5 [19]. The other sections from the clean colon lesion were kept at −80°C for further analysis.

### 2.8. Measurement of MPO, SOD, MDA, NO, and Proinflammatory Cytokine in Colonic Tissues

The activities of MPO, SOD, MDA, and NO were detected with the corresponding test kits for the supernatants of the colonic tissues. The levels of TNF-*α*, IL-1*β,* and IL-6 of the colonic tissues were measured using ELISA assay kits.

### 2.9. Statistical Analysis

Results were presented as mean ± SD (standard deviation) using SPSS 20.0 software. Statistical differences were carried out by one-way ANOVA followed by Dunnett's test. *P* values < 0.05 were considered statistically significant.

## 3. Results

### 3.1. UPLC-ESI-Q-TOF-MS/MS Profiles of GFE

**Figure 1:**
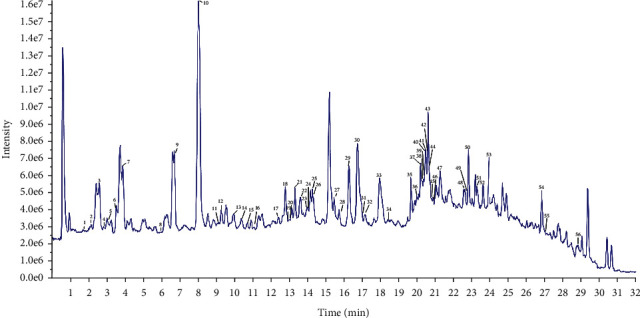
Total ion flow chromatogram of GFE in the negative ion mode by UPLC-Q-TOF/MS.

The components of GFE were analysed by UPLC-ESI-Q-TOF/MS. A total of 56 components were identified based on literatures and the detailed mass spectrometry data, while Table 2 shows the retention times, proposed formula, MS and fragments, and the identification results for the peaks labeled in the total ion flow chromatogram of GFE (Figure 1) [20, 21]. The components were mainly categorized into six structural groups, including iridoids, triterpenes, flavonoids, carotenoids, organic acids, and monoterpenoids.

### 3.2. Effects of GFE on DAI

**Figure 2:**
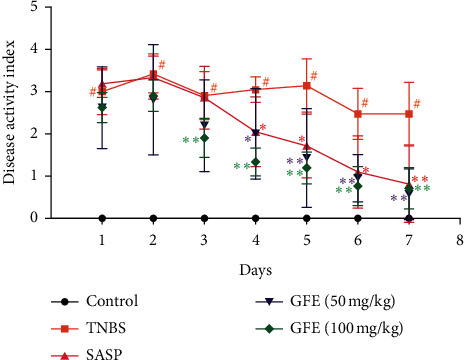
Effect of different doses of GFE (50 mg/kg, 100 mg/kg) and SASP (500 mg/kg) on DAI scores of TNBS-induced colitis in rats. Data are showed as mean ± SD. ^#^*p* < 0.01 *vs.* control group; ^*∗*^*p* < 0.05 and ^*∗∗*^*p* < 0.01 *vs.* TNBS group.

DAI score is a notable index to assess the severity of colitis. As shown in Figure 2, compared with the control group, the DAI score was significantly increased in TNBS group (*p* < 0.01) from Day 1, and treatment with GFE (50 mg/kg, 100 mg/kg) caused a significant decrease in the DAI score compared to the TNBS group at Day 3 and Day 4, respectively.

### 3.3. Macroscopic Changes

**Figure 3:**
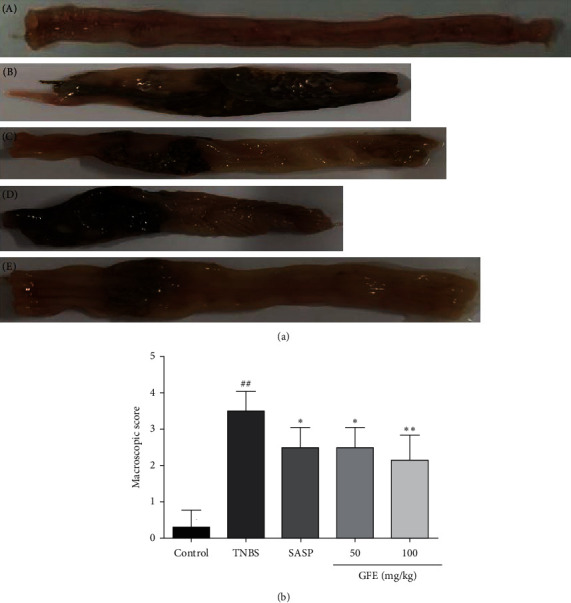
Effect of different doses of GFE (50 mg/kg, 100 mg/kg) and SASP (500 mg/kg) on colonic macroscopic appearance changes (A) and macroscopic score (B): (a) control group; (b) TNBS group; (c) SASP group; (d) GJEF treatment group, 50 mg/kg; (e) GJEF treatment group, 100 mg/kg. Data are showed as mean ± SD. ^##^*p* < 0.001 *vs.* control group; ^*∗*^*p* < 0.05 and ^*∗∗*^*p* < 0.01 *vs.* TNBS group.

Macroscopic changes are major indicators to evaluate the severity of colitis observed in rats administered with TNBS. In this study, macroscopic inspection of the colons displayed the presence of severe ulceration, edema, and tissue necrosis in TNBS group. In contrast, SASP and GFE (50 mg/kg, 100 mg/kg) treatment caused a remarkable loss in the macroscopic lesion in colonic tissue (Figure 3(a)). The corresponding macroscopic scores were also presented in Figure 3(b). The macroscopic score of TNBS group was dramatically increased compared with the control group (3.50 ± 0.55 vs. 0.29 ± 0.49, *p* < 0.01). This indicated that the model was successfully established. Treatment with SASP or GFE (50 mg/kg, 100 mg/kg) significantly decreased macroscopic sores compared with TNBS group.

### 3.4. Histological Changes

**Figure 4:**
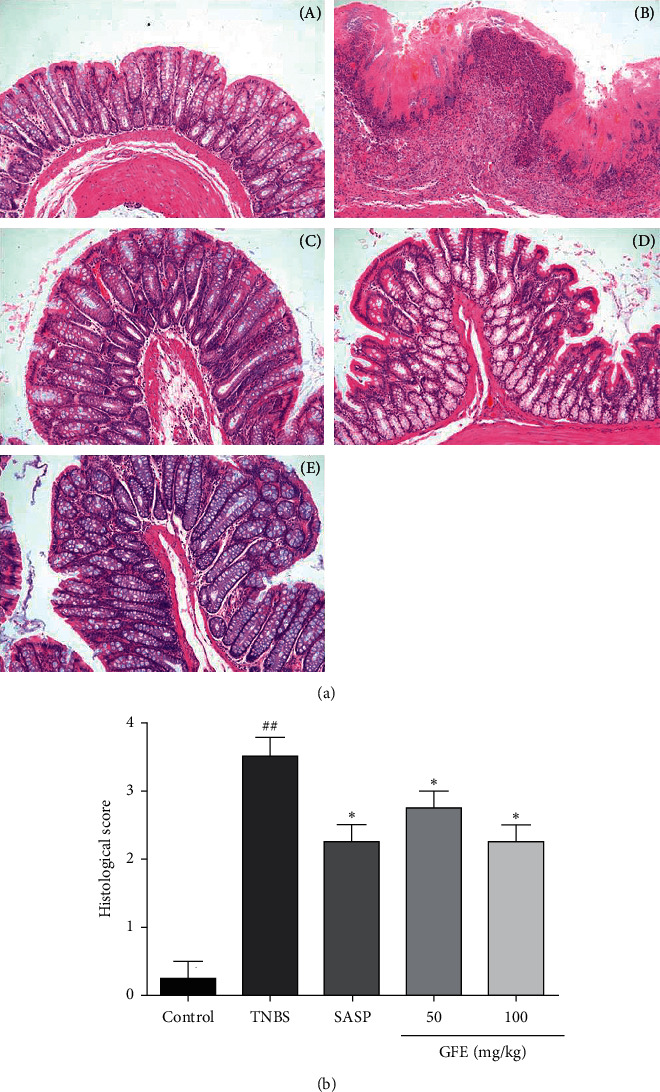
Effect of different doses of GFE (50 mg/kg, 100 mg/kg) and SASP (500 mg/kg) on colonic histological changes (A) and histological scores (B): (a) control group; (b)TNBS group; (c) SASP group; (d) GFE treatment group, 50 mg/kg; (e) GFE treatment group, 100 mg/kg. Data are showed as mean ± SD. ^##^*p* < 0.001 *vs.* control group; ^*∗*^*p* < 0.05 and ^*∗∗*^*p* < 0.01 *vs.* TNBS group. H&E straining, magnification 100×.

The histological study of colonic tissue showed inflammatory cell infiltration in the mucosa, loss of goblet cells, and epithelium and deformed or disappeared crypt in the TNBS group. The histologic sections of GFE (50 mg/kg, 100 mg/kg) or SASP groups showed moderate goblet cell loss, improvement of the crypt architecture, and mild inflammatory cell infiltration compared to those of the TNBS group (Figure 4(a)). The histological scores of GFE (50 mg/kg, 100 mg/kg) or SASP were also significantly decreased compared to the TNBS group (Figure 4(b)).

### 3.5. Effects of GFE on the MPO, SOD, MDA, and NO Levels

**Figure 5:**
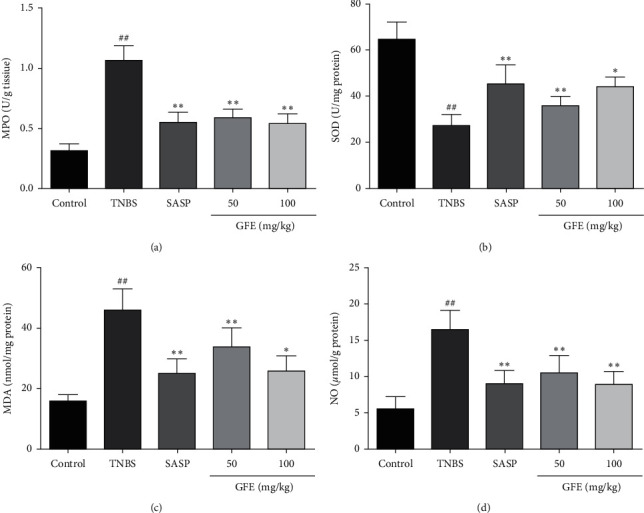
Effect of different doses of GFE (50 mg/kg, 100 mg/kg) and SASP (500 mg/kg) on MPO (a), SOD (b), MDA (c), and NO (d). Data are showed as mean ± SD. ^##^*p* < 0.001 *vs.* control group; ^*∗*^*p* < 0.05 and ^*∗∗*^*p* < 0.01 *vs.* TNBS group.

Treatment of rats with TNBS caused a significant increase in the colonic MPO levels (1.07 ± 0.12 U/g vs. 0.32 ± 0.057 U/g, *p* < 0.01) compared with that observed in the control group, and the colonic MPO levels were remarkably decreased (*p* < 0.01) by the treatment of 500 mg/kg SASP (0.55 ± 0.085 U/g), 50 mg/kg (0.59 ± 0.073 U/g), and 100 mg/kg GFE (0.54 ± 0.078 U/g), respectively (Figure 5(a)).

The SOD levels in the TNBS group were dramatically decreased in comparison to the control group (27.12 ± 4.86 U/mg vs. 64.59 ± 7.59 U/mg, *p* < 0.01). Treatment with SASP and GFE (50 mg/kg, 100 mg/kg) increased significantly the level of SOD in the colon (45.17 ± 8.36 U/mg, 35.81 ± 4.25 U/mg, and 43.98 ± 4.37 U/mg, *p* < 0.05, *p* < 0.01; Figure 5(b)). Compared to the control group, higher level of colonic MDA was found in TNBS group (15.83 ± 2.26 nmol/mg *vs.* 45.95 ± 7.04 nmol/mg, *p* < 0.01). The MDA level was decreased to 25.08 ± 4.93 nmol/mg, 33.70 ± 6.43 nmol/mg, and 25.86 ± 5.04 nmol/mg after treatment with SASP and GFE (50 mg/kg, 100 mg/kg) (Figure 5(c)). The higher colonic level of NO was in the TNBS group in comparison with the control group (16.48 ± 2.64 *μ*mol/g *vs.* 5.55 ± 1.78 *μ*mol/g, *p* < 0.01). The colonic NO level was significantly decreased by treatment with SASP and different doses of GFE (9.02 ± 1.82 *μ*mol/g, 10.48 ± 2.43 *μ*mol/g, and 8.88 ± 1.84 *μ*mol/g, *p* < 0.01, Figure 5(d)).

### 3.6. Effects of GFE on the Proinflammatory Cytokines

**Figure 6:**
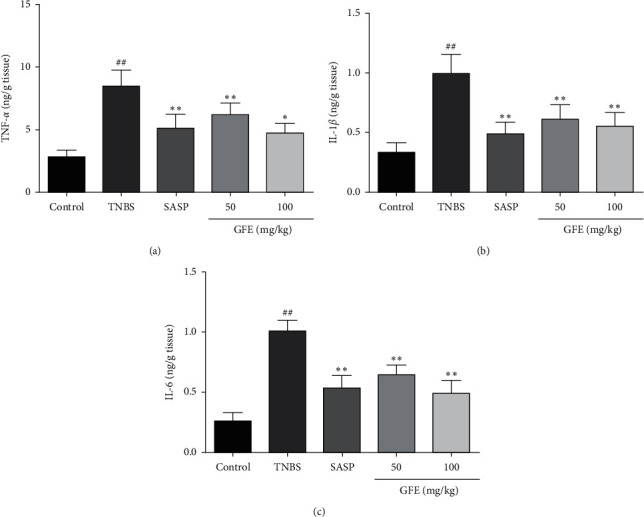
Effect of different doses of GFE (50 mg/kg, 100 mg/kg) and SASP (500 mg/kg) on TNF-*α* (a), IL-1*β* (b), and IL-6 (c). Data are showed as mean ± SD. ^##^*p* < 0.001 *vs.* control group; ^*∗*^*p* < 0.05 and ^*∗∗*^*p* < 0.01 *vs.* TNBS group.

The levels of TNF-*α*, IL-1*β,* and IL-6*β* were dramatically increased in the TNBS group compared with the control group (8.48 ± 1.30 ng/g vs. 2.82 ± 0.54 ng/g, 0.99 ± 0.17 ng/g vs. 0.33 ± 0.078 ng/g, 1.00 ± 0.089 ng/g vs. 0.26 ± 0.068 ng/g, *p* < 0.01). Conversely, the colonic TNF-*α*, IL-1*β,* and IL-6*β* levels were notably decreased in both GFE (50 mg/kg, 100 mg/kg) and SASP groups in comparison to the TNBS group (*p* < 0.05, *p* < 0.01; Figures 6(a)–6(c)).

## 4. Discussion

UC is a chronic inflammatory bowel disease which affects a million people around the world. Current treatment of UC is generally anti-inflammatory or immunosuppressive agents. However, most of these agents have many side effects and are proved to be inadequate for treatment. Thus, more and more natural products, especially medicinal plants, are considered as alternative treatments. GFE has been considered to have anti-inflammatory effect in previous studies. Therefore, we focused on the effect of GFE on TNBS-induced colitis in rats in the current study. TNBS-induced colitis model is one of the most appropriate models similar to human colitis in previous studies [1]. On this estimated model, DAI, macroscopic and histological changes were assessed in this study. GFE could reduce the DAI score and attenuate the macroscopic damage significantly induced by TNBS. The results of histological evaluation were consistent with macroscopic data, indicating that GFE treatment dramatically attenuated the destruction of the colonic tissue and its related inflammatory changes induced by TNBS. All these results above demonstrated that GFE had a noticeably protective effect against TNBS-induced colitis in rats. This efficacy was further proved by the investigation of biochemical and inflammatory biomarkers in colonic tissues.

It is known that reactive oxygen species (ROS) are involved in the initiation and progression of ulcerative colitis. Under normally physiological conditions, amounts of antioxidant systems control ROS. Nevertheless, excessive ROS released in inflammatory colitis lesions induce oxidative stress [22, 23]. MDA is an important oxidative stress indicator which is produced by lipid peroxidation. It was documented that the tissue level of MDA was increased in TNBS-induced colitis, which can also be observed in our study [24]. Meanwhile, the intestinal cells possess more efficient antioxidant defense to reduce the harmful effects of oxidative stress. SOD is a crucial antioxidant enzyme which could reduce oxidative damage and scavenge free radicals. Several studies also showed that the colonic SOD level was decreased in the colitis model [25, 26]. The change trends of colonic MDA and SOD levels in model rats were consistent with previous literatures, which indicated that the colitis model was established in our experiments successfully. GFE administrations (50 mg/kg, 100 mg/kg) significantly decreased the concentration of MDA and increased the activity of SOD. These findings indicated that amelioration of colon damage with GFE at least partly was related to the reduction of oxidative stress.

NO is a free gaseous signaling molecule that is synthesized by inducible nitric oxide synthase. Previous studies have shown that overproduction of NO leads to colonic mucosal damage and implicates in the pathogenesis of colitis [27, 28]. Hence, NO is an important indicator in inflammatory bowel disease. Meanwhile, the activity of MPO is also an important biomarker of neutrophils infiltration into the inflamed colonic tissue, which is commonly used to evaluate the severity degree of colonic inflammation in kinds of experimental studies and clinical trials [29, 30]. In this study, the colonic MPO and NO levels were increased in TNBS group, revealing that consequent inflammation and recruitment occurred in rats induced by TNBS. Treatment with GFE showed remarkably decreased levels of MPO and NO in colonic tissue, thus indicating the effect of GFE treatment was associated with the anti-inflammatory property.

Proinflammatory cytokines such as TNF-*α*, 1L-1*β*, and 1L-6 have essential roles in the pathological development of UC [31]. As previous studies demonstrated, the proinflammatory cytokine levels were increased in UC patients and TNBS-induced colitis rats model [32]. TNF-*α* could recruit leukocytes from the inflamed area and stimulate the expression of cytokines which is a key target molecule of taking part in intestinal inflammation [33, 34], and 1L-1*β* and 1L-6 are also important indicators in the progression of UC. IL-1*β* could stimulate the recruitment of neutrophils to the inflamed colonic tissue [35]. IL-6 plays an important role in enhancing T-cell survival and apoptosis resistance in the lamina propria at the inflamed site [36, 37]. Therefore, targeting proinflammatory cytokines had become a therapeutic approach for UC patients. In the current study, treatment with GFE significantly decreased the colonic TNF-*α*, 1L-1*β*, and 1L-6 levels. These findings also indicated that GFE may influence the production of cytokines and inhibit immune inflammation.

Furthermore, this study explored the effective components in GFE by UPLC-ESI-Q-TOF/MS. 56 compounds of six main structural types in GFE, including iridoids, flavonoids, triterpenes, monoterpenoids, carotenoids, and phenolic acids were identified based on UPLC-ESI-QTOF/MS analysis. According to previous studies, iridoids-type compound (e.g., geniposide and genipin), carotenoids-type compound (e.g., crocin), and phenolic acid-type compound (e.g., chlorogenic acid and gallic acid) have been demonstrated to exhibit good antioxidant activity and anti-inflammatory activity [38–42]. Geniposide and chlorogenic acid had even been proved to treat UC in animal models through inflammatory pathways. Hence, the effect of the type of iridoids, carotenoids, and phenolic acid compounds from GFE on ulcerative colitis will be investigated in our further research.

## 5. Conclusion

Based on these findings of the current study, treatment with GFE could ameliorate TNBS-induced colitis in rats, and this protective effect of GFE may be mediated through its anti-inflammatory and antioxidative properties. GFE could be a potential agent for colitis that deserves further investigation.

## Figures and Tables

**Table 1:** Histopathological scoring pattern in colon tissue.

Histopathological scoring	Microscopic colon damage
0	Normal colonic tissue
1	Inflammation or focal ulceration limited to the mucosa
2	Focal or extensive ulceration and inflammation limited to the mucosa and the submucosa
3	Focal or extensive ulceration and inflammation limited with involvement of the muscularis propria
4	Focal or extensive ulceration and inflammation limited with involvement of the serosa
5	Extensive ulceration and transmural inflammation with involvement of the serosa

**Table 2:** Structure of the 56 compounds detected in GFE.

No.	*t* (min)	Mass (m/*z*)	Calculated ass (m/*z*)	Proposed formula	Fragments	Deduced structure	Type
1	1.77	389.1077	389.1084	C16H22O11	227, 183, 165, 139	Deacetyl asperulosidic acid	Iridoids
2	2.13	373.1130	373.1135	C16H22O10	193,149,123,167,121,101,211	Gardoside	Iridoids
3	2.58	391.1237	391.1240	C16H24O11	229, 211, 193, 185, 167, 149	Shanzhiside	Iridoids
4	2.64	373.1127	373.1135	C16H22O10	149, 123, 105, 211	Geniposidic acid	Iridoids
5	3.28	449.1286	449.1295	C17H24O11	241, 127, 223, 193, 101	Scandoside methyl ester	iridoids
6	3.53	449.1288	449.1295	C17H24O11	241, 127, 223, 193, 101	Gardenoside	Iridoids
7	3.86	345.1552	345.1549	C16H26O8	165, 121, 183, 101, 89	Jasminoside B	Monoterpenoids
8	6.17	353.0872	353.0873	C16H18O9	191, 135	Chlorogenic acid	Organic acids
9	6.64	549.1800	549.1820	C_23_H_34_O_15_	225, 123, 101, 207	Genipin-1-*β*-D-gentiobioside	Iridoids
10	8.02	387.1282	387.1291	C17H24O10	225, 123, 101, 207	Geniposide	Iridoids
11	9.28	375.1653	375.1655	C17H27O9	113, 89, 101, 59, 85, 71, 143	Jasminoside A/*E*	Monoterpenoids
12	9.55	375.1650	375.1655	C17H27O9	113, 89, 101, 59, 85, 71, 143	Jasminoside A/*E*	Monoterpenoids
13	9.95	183.1039	183.1021	C10H16O3	139, 137, 109, 123	Jasminodiol	Monoterpenoids
14	10.46	351.1423	351.1420	C16H24O7	167	Zataroside B	Monoterpenoids
15	10.69	503.1747	503.1765	C22H32O13	205, 223, 190	2-Methyl-L-erythritol-4-O-(6-O-trans-sinapoyl)-*β*-D-glucopyranoside	Organic acids
16	11.29	503.1747	503.1765	C22H32O13	205, 223, 190	2-Methyl-L-erythritol-1-O-(6-O-trans-sinapoyl)-*β*-D-glucopyranoside	Organic acids
17	12.64	609.1437	609.1456	C27H30O16	301, 271, 343	Quercetin-3-O-rutinoside	Flavonoids
18	12.76	429.2111	429.2122	C19H26O11	191, 209, 339, 113, 249	10-O-Acetylgeniposide	Iridoids
19	12.79	609.1430	609.1456	C27H30O16	301, 300, 151, 179	Rutin	Flavonoids
20	13.13	463.0876	463.0863	C21H20O12	300, 301, 271, 151, 255	Isoquercitrin	Flavonoids
21	13.30	581.2234	581.2234	C28H38O13	401, 389, 371, 356, 265	(+)-Lyoniresinol-3a-O-b-glucopyranoside	Lignans
22	13.58	579.1695	579.1714	C27H32O14	205, 325, 367, 385, 223, 123	6′-O-trans-Sinapoyl gardoside	Iridoids
23	13.81	539.1177	515.1190	C25H24O12	353, 173, 179, 191	3,4-Di-O-Caffeoylquinic acid/3,5-Di-O-caffeoylquinic acid	Phenolic acids
24	13.91	565.1903	565.1921	C27H34O13	325, 265, 223, 295	11-(6-O-trans-Sinapoylglucopyranosyl)-gardendiol	Iridoids
25	14.25	539.1167	515.1190	C25H24O12	353, 173, 179, 191	3,4-Di-O-Caffeoylquinic acid/3,5-Di-O-caffeoylquinic acid	Phenolic acids
26	14.29	491.2109	491.2129	C22H36O12	167, 323, 221, 125, 263	Jasminoside S/H/I	Monoterpenoids
27	15.45	755.2377	755.2399	C34H44O19	123, 223, 205, 101, 427,	6′-O-trans-Sinapoyl genipin gentiobioside	Iridoids
28	15.73	725.2278	725.2293	C33H42O18	499, 123, 193, 225	6′′-O-trans-Feruloyl-genipin gentiobioside	Iridoids
29	16.30	659.15969	659.1612	C31H32O16	497, 335, 353, 191, 161	3,4-Dicaffeoyl-5-(3-hydroxy-3-methylglutaroyl) quinic acid	Phenolic acids
30	16.76	975.3686	975.3709	C44H64O24	651, 327, 283, 239	trans-Crocin4/cis-crocin4	Carotenoids
31	16.90	551.2115	551.2129	C27H36O12	533, 521, 265, 367	6′-O-trans-Sinapoyl jasminoside L	Monoterpenoids
32	17.16	559.1433	559.1452	C27H28O13	173, 223,397	5-O-Caffeoyl-4-O-sinapoylquinic acid	Phenolic acids
33	17.97	559.1432	559.1452	C27H28O13	173, 223,397	4-Sinapoyl-5-caffeoylquinic acid	Phenolic acids
34	18.12	593.1845	593.1870	C28H34O14	367, 223, 205, 123	6′- trans-Sinapoyl geniposide	Iridoids
35	19.68	813.3158	813.3181	C38H54O19	651, 327, 283	trans-Crocin3/cis-crocin3	Carotenoids
36	20.01	301.0351	301.0348	C15H10O7	151, 273, 73	Quercetin	Flavonoids
37	20.19	679.2216	679.2238	C32H40O16	531, 225, 123, 147, 101	6”-O-trans-p-Cinnamoyl genipin gentiobioside	Iridoids
38	20.28	345.0611	345.0610	C17H14O8	315, 330, 287	5,7,3,4-Tetrahydroxy-6,8-dimethoxy flavone	Flavonoids
39	20.37	533.2006	533.2023	C27H34O11	205, 367, 223, 165, 190	6′-O-trans-Sinapoyl jasminoside A	Monoterpenoids
40	20.44	975.368	975.371	C44H64O24	651, 327, 283, 239	trans-Crocin4/cis-crocin4	Carotenoids
41	20.45	651.2630	651.2653	C32H44O14	327, 283, 239	cis-Crocin2/trans-crocin2/cis-crocin2/trans-crocin2′	Carotenoids
42	20.54	533.20063	533.2023	C27H34O11	205, 367, 223, 165, 190	6′-O-trans-Sinapoyl jasminoside C	Monoterpenoids
43	20.65	651.26332	651.2653	C32H44O14	327, 283, 239	cis-Crocin2/trans-crocin2/cis-crocin2/trans-crocin2′	Carotenoids
44	20.70	813.3158	813.3181	C38H54O19	651, 327, 283	trans-Crocin3/cis-crocin3	Carotenoids
45	20.80	375.0710	375.0716	C18H16O9	360, 345, 330	4,5,6,7-Tetrahydroxy-3,3,5-trimethoxyflavone	Flavonoids
46	20.89	329.0661	329.0661	C17H13O7	314, 299, 271, 227	2-(3,5-Dihyroxy-4-ethoxyphenyl)-5-hydroxy-7-methoxy-4H-chromen-4-one	Flavonoids
47	21.29	651.2632	651.2653	C32H44O14	327, 283, 239	cis-Crocin2/trans-crocin2/cis-crocin2/trans-crocin2′	Carotenoids
48	22.56	499.3035	499.3060	C30H44O6	455, 437, 481, 393	Dikamaliartanes A	Triterpenes
49	22.79	327.1596	327.1596	C20H24O4	239, 283	Crocetin	Carotenoids
50	22.82	487.3405	487.3424	C30H48O5	469, 437, 425, 393	Erubigenin	Triterpenes
51	23.28	489.3558	489.3580	C30H50O5	471, 441	Secaubrytriol	Triterpenes
52	23.61	269.0453	269.0450	C15H10O5	241, 225	Genistein	Flavonoids
53	23.97	485.3249	485.3267	C30H46O5	441, 423, 467, 367	Gardenic acid B	Triterpenes
54	26.87	471.3458	471.3474	C30H48O4	453, 423, 409	Quadrangularic acid E	Triterpenes
55	26.93	469.3302	469.3318	C30H46O4	451, 407, 423	9,19-Cyclolanost-24-ene-3,23-dione	Triterpenes
56	28.67	455.3515	455.3525	C30H48O3	407, 391, 377	Ursolic acid/oleanolic acid	Triterpenes

## Data Availability

The data used to support the findings of this study are available from the corresponding author upon request.
